# 6-[3-(*p*-Tolyl­sulfonyl­amino)­prop­yl]diquino­thia­zine^1^

**DOI:** 10.1107/S1600536813013950

**Published:** 2013-05-25

**Authors:** Małgorzata Jeleń, Aleksander Shkurenko, Kinga Suwińska, Krystian Pluta, Beata Morak-Młodawska

**Affiliations:** a Department of Organic Chemistry, The Medical University of Silesia, ul. Jagiellońska 4, PL–41 200 Sosnowiec, Poland; b Institute of Physical Chemistry, Polish Academy of Sciences, ul. Kasprzaka 44/52, PL–01 224 Warsaw, Poland; c Faculty of Biology and Environmental Sciences, Cardinal Stefan Wyszynski University, ul. Wóycickiego 1/3, PL–01 938, Warszawa, Poland

## Abstract

In the title mol­ecule {systematic name: *N*-[3-(diquino[3,2-*b*;2′,3′-*e*][1,4]thia­zin-6-yl)prop­yl]-4-methyl­benzene­sulfon­amide}, C_28_H_24_N_4_O_2_S_2_, the penta­cyclic system is relatively planar [maximum deviation from the mean plane = 0.242 (1) Å]. The dihedral angle between two quinoline ring systems is 8.23 (2)° and that between the two halves of the 1,4-thia­zine ring is 5.68 (3)°. The conformation adopted by the 3-(*p*-tolyl­sulfonyl­amino)­propyl substituent allows for the formation of an intra­molecular N—H⋯N hydrogen bond and places the benzene ring of this substituent above one of the quinoline fragments of the penta­cyclic system. In the crystal, mol­ecules are arranged *via* π–π stacking inter­actions into (0-11) layers [centroid–centroid distances = 3.981 (1)–4.320 (1) Å for the rings in the penta­cyclic system and 3.645 (1) Å for the tolyl benzene rings]. In addition, mol­ecules are involved in weak C—H⋯O, which connect the layers, and C—H⋯S hydrogen bonds. The title compound shows promising anti­cancer activity against renal cancer cell line UO-31.

## Related literature

For the structures of hetero­penta­cenes, see: Anthony (2006 ▶); Isaia *et al.* (2009 ▶); Yoshida *et al.* (1994 ▶). For recent literature on the biological activity of pheno­thia­zines, see: Aaron *et al.* (2009 ▶); Pluta *et al.* (2011 ▶). For the synthesis and biological activity of 6-substituted diquino­thia­zines, see: Nowak *et al.* (2007 ▶); Jeleń & Pluta (2009 ▶); Pluta *et al.* (2010 ▶). For crystal structures of pheno­thia­zines, see: Chu (1988 ▶). For information on aza­pheno­thia­zines, their nomenclature and synthesis, see: Pluta *et al.* (2009 ▶). For hetero­penta­cenes with quinoline moieties containing nitro­gen, sulfur, oxygen and selenium, see: Nowak *et al.* (2002 ▶); Pluta *et al.* (2000 ▶).
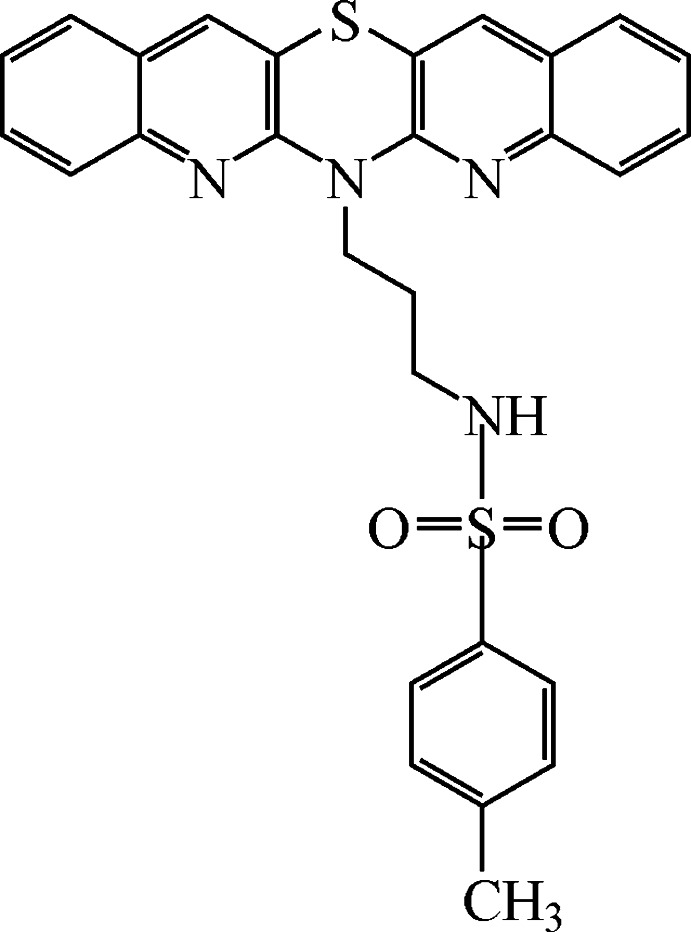


## Experimental

### 

#### Crystal data

C_28_H_24_N_4_O_2_S_2_
*M* *_r_* = 512.63
Triclinic, 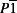

*a* = 9.3986 (3) Å
*b* = 10.3793 (3) Å
*c* = 12.6207 (3) Å
α = 80.735 (2)°
β = 81.959 (2)°
γ = 77.999 (2)°
*V* = 1181.30 (6) Å^3^
*Z* = 2
Mo *K*α radiation
μ = 0.26 mm^−1^
*T* = 100 K
0.39 × 0.34 × 0.23 mm

#### Data collection

Agilent SuperNova Dual (Cu at zero, Eos) diffractometer
Absorption correction: analytical [*CrysAlis PRO* (Agilent, 2012 ▶) and Clark & Reid (1995 ▶)] *T*_min_ = 0.937, *T*_max_ = 0.965
24091 measured reflections
7396 independent reflections
6770 reflections with *I* > 2σ(*I*)
*R*_int_ = 0.015

#### Refinement

*R*[*F* ^2^ > 2σ(*F* ^2^)] = 0.033
*wR*(*F* ^2^) = 0.093
*S* = 1.04
7396 reflections
329 parameters
H atoms treated by a mixture of independent and constrained refinement
Δρ_max_ = 0.42 e Å^−3^
Δρ_min_ = −0.40 e Å^−3^

### 

Data collection: *CrysAlis PRO* (Agilent, 2012 ▶); cell refinement: *CrysAlis PRO*; data reduction: *CrysAlis PRO*; program(s) used to solve structure: *SHELXS97* (Sheldrick, 2008 ▶); program(s) used to refine structure: *SHELXL97* (Sheldrick, 2008 ▶); molecular graphics: *ORTEPIII* (Burnett & Johnson, 1996 ▶) and *Mercury* (Macrae *et al.*, 2008 ▶); software used to prepare material for publication: *publCIF* (Westrip, 2010 ▶).

## Supplementary Material

Crystal structure: contains datablock(s) I, global. DOI: 10.1107/S1600536813013950/gk2566sup1.cif

Structure factors: contains datablock(s) I. DOI: 10.1107/S1600536813013950/gk2566Isup2.hkl

Supplementary material file. DOI: 10.1107/S1600536813013950/gk2566Isup3.cml

Additional supplementary materials:  crystallographic information; 3D view; checkCIF report

## Figures and Tables

**Table 1:** Hydrogen-bond geometry (Å, °)

*D*—H⋯*A*	*D*—H	H⋯*A*	*D*⋯*A*	*D*—H⋯*A*
N18—H18⋯N5	0.876 (14)	2.243 (15)	2.9973 (12)	144.1 (12)
C15—H15*A*⋯O20^i^	0.99	2.33	3.1641 (12)	141
C11—H11⋯O21^ii^	0.95	2.55	3.3933 (13)	149
C15—H15*B*⋯S13^iii^	0.99	2.86	3.6912 (11)	142
C17—H17*B*⋯O21^iv^	0.99	2.47	3.3013 (13)	141

Symmetry codes: (i) 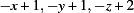; (ii) 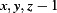; (iii) 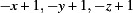; (iv) 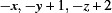.

**Fig. 1.**
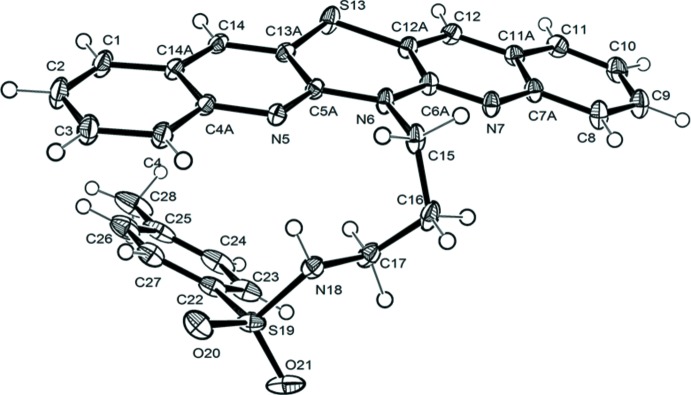
Molecular structure with displacement ellipsoids shown at the 50% probability level.

**Fig. 2.**
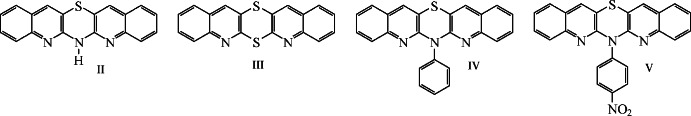
Related compounds.

**Fig. 3.**
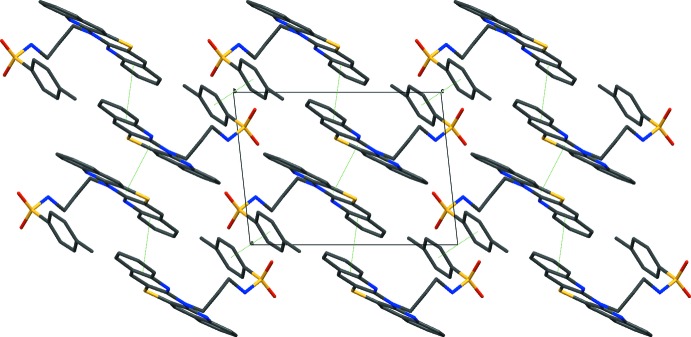
Structure of the (0 -1 1) layer: the π–π stacking interactions between pentacyclic systems are 3.589 (1) and 3.841 (1) Å [distance measured between r.m.s. planes of fully eclipsed and partially eclipsed benzene rings of pentacyclic systems, respectively] and 3.453 (1) Å between r.m.s. planes of benzene rings of toluene substituents.

## supplementary crystallographic information

### Comment

Heteropentacenes, mainly aza-, thia- and azathiapentacenes, are considered as polyheterocyclic donor molecules to form organic semiconductors (Anthony, 2006). We obtained heteropentacenes containing nitrogen, sulfur, oxygen and selenium *via* the annulation reactions (Nowak *et al.*, 2002). One of this type was 6*H*-5,6,7-triaza-13-thiapentacene (II, Figure 2) which can be regarded as a new modified phenothiazine system, pentacyclic diquinothiazine, where two quinoline rings were incorporated in the ring system instead of benzene rings. This compound was further transformed by introduction of 6-alkyl, aryl, heteroaryl and aminoalkyl substituents at the thiazine nitrogen atoms. It is well known that neuroleptic phenothiazines have tricyclic dibenzothiazine ring system and the aminoalkyl substituent at the thiazine nitrogen atom in position 10. All these compounds are folded and have the central thiazine ring in a boat conformation with the aminoalkyl group in the equatorial position. 6-Substituted diquinothiazines with the aminoalkyl groups and their acyl and sulfonyl derivatives exhibit promising anticancer activities against the cell lines of 9 types of human cancer: leukemia, melanoma, non-small cell lung cancer, colon cancer, *CNS* cancer, ovarian cancer, renal cancer, prostate cancer and breast cancer (Pluta *et al.*, 2010). None of phenothiazines or azaphenothiazine with the *p*-tolylsulfonylaminopropyl substituent have been examined by X-ray crystallography so far. The title compound (I) was obtained in a few step synthesis starting from the reactions of heteropentacenes, 6*H*-5,6,7-triaza-13-thiapentacene (II) and 5,7-diaza-6,13-dithiapentacene (III), with appropriate reagents (see Experimental). The structure of the title compound was assigned by spectroscopic (^1^H NMR and MS) analysis. The diquinothiazine structure of C_2v_ symmetry shows a lack of the Smiles rearrangement during thiazine ring closure (Jeleń & Pluta, 2009). X-ray analysis fully confirmed this structure as 6-[3(*p*-tolylsulfonylamino)propyl]diquino[3,2 - b;2',3'-e][1,4]thiazine. All the classical neuroleptic phenothiazines are folded along the N–S axis with the dihedral angle of 134.0–153.6° and with the aminoalkyl group in equatorial positions (Chu, 1988). The title molecule is close to planar with the dihedral angle between the quinoline rings of 171.77 (2)° and the angle between two halves of the thiazine ring of 174.32 (3)°. The S13···N6–C15 angle is 176.70 (6)°. The endocyclic bond angles at heteroatoms in the central ring (C12A–S13–C13A and C5A–N6–C6A) are quite large, 102.56 (4)° and 125.04 (8)° in accord with the thiazine ring flat conformation. It is interesting that the planarity of the triazathiapentacene system is dependent on the substituent nature at the thiazine nitrogen atom. When the substituent was an electron-donating group (phenyl in compound IV; Pluta *et al.*, 2000), the ring system was folded, when electron-deficient ( *p*-nitrophenyl group in compound V; Nowak *et al.*, 2007) the system was almost planar. The bonds around the sulfur atom S19 of the sulfonamide group show a distortion from tetrahedral geometry. Whereas the O22—S19—O21 bond angle is 119.98 (5)°, three other O–S19–*X* (N18 or C22) bond angles are 106.68 (4)–107.48 (5)°. The bonds around thiazine N6 and sulfonamide N18 atoms show planar and pyramidal arrangement, respectively, as shown by the sum bond angles (359.97 (8)° and 340.25 (9)°, repectively). The *p*-tolylsulfonylaminopropyl substituent is not coplanar with pentacyclic ring system and shows unexpectedly U-conformation with the benzene ring placed over the pentacene system, with the dihedral angle between the C22–C26 and N6/C5A/C6A/C12A/C13A/S13 planes of 30.15 (3)°. The torsion angles including the propyl group (C15–C16–C17) show the synclinal/synclinal arrangement of the carbon chain. The torsion angles involving the sulfonamide group (C17–N18–S19–C22) show antiperiplanar/synclinal/synclinal arrangement. There is intramolecular N18–H18···N5 hydrogen bond which stabilizes the U shape of the *p*-tolylsulfonylaminopropyl substituent. Three intermolecular C–H···O hydrogen bonds involving the sulfonamide group and one C–H···S hydrogen bond involving the thiazine sulfur atom exist in the crystal. The molecules which are related *via* centers of symmetry at 0,1/2,1/2 and 1/2,1/2,1/2 stack in ribbons along the *a* direction *via*π–π interactions of the pentacyclic systems. The ribbons are further arranged into (0 -1 1) layers *via* another π–π interactions between benzene rings of toluene substituents (Figure 3). Layers are glued together by the mentioned above C–H···O hydrogen bonds between aromatic carbon atoms of pentacyclic systems of one layer and sulfonamide oxygen atoms of the neighbouring layer.

The title compound shows promising anticancer activity against renal cancer cell line UO-31.

### Experimental

The title compound was obtained in a few step synthesis as described by Jeleń *et al.* (2009) starting from the reactions of 6*H*-5,6,7-triaza-13-thiapentacene (II) with phthalimidopropyl bromide (followed by hydrolysis) or 5,7-diaza-6,13-dithiapentacene (III) with 1,3-diaminopropane to obtain aminopropyldiquinothiazine (IV). Compound (IV) was sulfonylated with *p*-toluenesulfonyl chloride. The title compound has melting point 439–440 K. X-ray quality crystals were grown from chloroform-ethanol mixture by slow evaporation.

### Refinement

All H atoms were treated as riding atoms in geometrically calculated positions, with d(C–H) = 0.95, 0.99 and 0.98 Å for aromatic, methylene and methyl hydrogens, respectively, except of the H atom in the N–H group of which positional parameters were refined freely, *U*_iso_(H) = *kU*_eq_(C,N), where *k* = 1.5 for the methyl group and *k* = 1.2 otherwise.

### Crystal data

**Table:**

C_28_H_24_N_4_O_2_S_2_	*Z* = 2
*M**_r_* = 512.63	*F*(000) = 536
Triclinic, *P*1	*D*_x_ = 1.441 Mg m^−^^3^
Hall symbol: -P 1	Mo *K*α radiation, λ = 0.71073 Å
*a* = 9.3986 (3) Å	Cell parameters from 16661 reflections
*b* = 10.3793 (3) Å	θ = 3.3–32.2°
*c* = 12.6207 (3) Å	µ = 0.26 mm^−^^1^
α = 80.735 (2)°	*T* = 100 K
β = 81.959 (2)°	Block, yellow
γ = 77.999 (2)°	0.39 × 0.34 × 0.23 mm
*V* = 1181.30 (6) Å^3^	

### Data collection

**Table:**

Agilent SuperNova Dual (Cu at zero, Eos) diffractometer	7396 independent reflections
Radiation source: SuperNova (Mo) X-ray Source	6770 reflections with *I* > 2σ(*I*)
Mirror monochromator	*R*_int_ = 0.015
Detector resolution: 16.2974 pixels mm^-1^	θ_max_ = 31.0°, θ_min_ = 3.3°
ω scans	*h* = −13→13
Absorption correction: analytical [*CrysAlis PRO* (Agilent, 2012) and Clark & Reid (1995)]	*k* = −15→15
*T*_min_ = 0.937, *T*_max_ = 0.965	*l* = −18→18
24091 measured reflections	

### Refinement

**Table:**

Refinement on *F*^2^	Primary atom site location: structure-invariant direct methods
Least-squares matrix: full	Secondary atom site location: difference Fourier map
*R*[*F*^2^ > 2σ(*F*^2^)] = 0.033	Hydrogen site location: inferred from neighbouring sites
*wR*(*F*^2^) = 0.093	H atoms treated by a mixture of independent and constrained refinement
*S* = 1.04	*w* = 1/[σ^2^(*F*_o_^2^) + (0.0463*P*)^2^ + 0.437*P*] where *P* = (*F*_o_^2^ + 2*F*_c_^2^)/3
7396 reflections	(Δ/σ)_max_ = 0.002
329 parameters	Δρ_max_ = 0.42 e Å^−^^3^
0 restraints	Δρ_min_ = −0.40 e Å^−^^3^

### Special details

**Table:**

Geometry. All e.s.d.'s (except the e.s.d. in the dihedral angle between two l.s. planes) are estimated using the full covariance matrix. The cell e.s.d.'s are taken into account individually in the estimation of e.s.d.'s in distances, angles and torsion angles; correlations between e.s.d.'s in cell parameters are only used when they are defined by crystal symmetry. An approximate (isotropic) treatment of cell e.s.d.'s is used for estimating e.s.d.'s involving l.s. planes.
Refinement. Refinement of *F*^2^ against ALL reflections. The weighted *R*-factor *wR* and goodness of fit *S* are based on *F*^2^, conventional *R*-factors *R* are based on *F*, with *F* set to zero for negative *F*^2^. The threshold expression of *F*^2^ > σ(*F*^2^) is used only for calculating *R*-factors(gt) *etc*. and is not relevant to the choice of reflections for refinement. *R*-factors based on *F*^2^ are statistically about twice as large as those based on *F*, and *R*- factors based on ALL data will be even larger.

### Fractional atomic coordinates and isotropic or equivalent isotropic displacement parameters (Å^2^)

**Table:**

	*x*	*y*	*z*	*U*_iso_*/*U*_eq_	
C1	0.49893 (13)	1.01160 (10)	0.71760 (9)	0.0272 (2)	
H1	0.4759	1.0911	0.6691	0.033*	
C2	0.55824 (14)	1.01426 (11)	0.81047 (10)	0.0316 (2)	
H2	0.5750	1.0958	0.8266	0.038*	
C3	0.59430 (13)	0.89597 (11)	0.88211 (10)	0.0284 (2)	
H3	0.6354	0.8987	0.9462	0.034*	
C4	0.57070 (11)	0.77664 (10)	0.86036 (8)	0.02243 (18)	
H4	0.5972	0.6975	0.9085	0.027*	
C4A	0.50694 (10)	0.77236 (9)	0.76632 (8)	0.01797 (16)	
N5	0.47657 (8)	0.65388 (8)	0.75030 (6)	0.01707 (14)	
C5A	0.41609 (9)	0.64626 (9)	0.66422 (7)	0.01604 (16)	
N6	0.38838 (9)	0.52185 (8)	0.65386 (7)	0.01928 (15)	
C6A	0.31384 (10)	0.49652 (9)	0.57300 (8)	0.01871 (17)	
N7	0.28831 (10)	0.37503 (8)	0.58262 (7)	0.02115 (16)	
C7A	0.21392 (11)	0.34230 (10)	0.50797 (8)	0.02097 (18)	
C8	0.18288 (13)	0.21269 (10)	0.52247 (9)	0.0270 (2)	
H8	0.2161	0.1500	0.5816	0.032*	
C9	0.10458 (14)	0.17702 (11)	0.45114 (10)	0.0307 (2)	
H9	0.0841	0.0897	0.4613	0.037*	
C10	0.05463 (14)	0.26913 (12)	0.36322 (9)	0.0301 (2)	
H10	−0.0011	0.2440	0.3154	0.036*	
C11	0.08566 (12)	0.39472 (11)	0.34607 (9)	0.0264 (2)	
H11	0.0536	0.4555	0.2856	0.032*	
C11A	0.16556 (11)	0.43355 (10)	0.41872 (8)	0.02102 (18)	
C12	0.19770 (11)	0.56268 (10)	0.40906 (8)	0.02207 (18)	
H12	0.1697	0.6267	0.3492	0.026*	
C12A	0.26858 (11)	0.59588 (9)	0.48515 (8)	0.01975 (17)	
S13	0.30095 (3)	0.75808 (3)	0.46855 (2)	0.02831 (7)	
C13A	0.37960 (10)	0.76078 (9)	0.58521 (7)	0.01722 (16)	
C14	0.40755 (10)	0.87994 (9)	0.60157 (8)	0.01950 (17)	
H14	0.3834	0.9565	0.5503	0.023*	
C14A	0.47201 (11)	0.89058 (9)	0.69388 (8)	0.01988 (17)	
C15	0.43639 (12)	0.40812 (9)	0.73615 (8)	0.02281 (19)	
H15A	0.5134	0.4294	0.7728	0.027*	
H15B	0.4803	0.3295	0.6994	0.027*	
C16	0.31325 (14)	0.37268 (10)	0.82111 (9)	0.0278 (2)	
H16A	0.2456	0.3360	0.7859	0.033*	
H16B	0.3561	0.3015	0.8761	0.033*	
C17	0.22489 (12)	0.48651 (11)	0.87824 (8)	0.02409 (19)	
H17A	0.1897	0.5623	0.8239	0.029*	
H17B	0.1383	0.4578	0.9222	0.029*	
S19	0.22842 (3)	0.62938 (3)	1.034198 (18)	0.02204 (6)	
O20	0.33837 (9)	0.65975 (8)	1.08936 (6)	0.02723 (16)	
O21	0.11287 (9)	0.56819 (11)	1.09446 (7)	0.0372 (2)	
C22	0.14699 (11)	0.77850 (11)	0.95968 (8)	0.02354 (19)	
C23	0.01915 (12)	0.78269 (14)	0.91344 (9)	0.0304 (2)	
H23	−0.0268	0.7075	0.9245	0.036*	
C24	−0.03909 (13)	0.89937 (15)	0.85096 (9)	0.0369 (3)	
H24	−0.1254	0.9030	0.8185	0.044*	
C25	0.02520 (14)	1.01107 (14)	0.83459 (9)	0.0362 (3)	
C26	0.15136 (14)	1.00516 (13)	0.88263 (9)	0.0333 (3)	
H26	0.1959	1.0811	0.8730	0.040*	
C27	0.21278 (12)	0.88873 (11)	0.94467 (9)	0.0267 (2)	
H27	0.2995	0.8849	0.9766	0.032*	
C28	−0.04257 (19)	1.13661 (16)	0.76733 (10)	0.0514 (4)	
H28A	−0.0395	1.1199	0.6927	0.077*	
H28B	0.0122	1.2069	0.7687	0.077*	
H28C	−0.1445	1.1646	0.7969	0.077*	
N18	0.31353 (9)	0.52970 (8)	0.94864 (7)	0.01995 (15)	
H18	0.3886 (16)	0.5609 (14)	0.9126 (12)	0.024*	

### Atomic displacement parameters (Å^2^)

**Table:**

	*U*^11^	*U*^22^	*U*^33^	*U*^12^	*U*^13^	*U*^23^
C1	0.0354 (5)	0.0178 (4)	0.0308 (5)	−0.0121 (4)	−0.0060 (4)	0.0007 (4)
C2	0.0432 (6)	0.0208 (5)	0.0365 (6)	−0.0153 (4)	−0.0099 (5)	−0.0036 (4)
C3	0.0364 (6)	0.0247 (5)	0.0287 (5)	−0.0130 (4)	−0.0091 (4)	−0.0036 (4)
C4	0.0260 (4)	0.0203 (4)	0.0232 (4)	−0.0087 (4)	−0.0054 (4)	−0.0016 (3)
C4A	0.0181 (4)	0.0161 (4)	0.0200 (4)	−0.0056 (3)	−0.0007 (3)	−0.0017 (3)
N5	0.0176 (3)	0.0150 (3)	0.0190 (3)	−0.0036 (3)	−0.0033 (3)	−0.0016 (3)
C5A	0.0156 (4)	0.0133 (4)	0.0186 (4)	−0.0022 (3)	−0.0027 (3)	−0.0006 (3)
N6	0.0259 (4)	0.0122 (3)	0.0209 (4)	−0.0029 (3)	−0.0114 (3)	0.0011 (3)
C6A	0.0212 (4)	0.0156 (4)	0.0197 (4)	−0.0018 (3)	−0.0078 (3)	−0.0010 (3)
N7	0.0264 (4)	0.0150 (3)	0.0237 (4)	−0.0026 (3)	−0.0111 (3)	−0.0019 (3)
C7A	0.0241 (4)	0.0182 (4)	0.0219 (4)	−0.0021 (3)	−0.0083 (3)	−0.0040 (3)
C8	0.0350 (5)	0.0179 (4)	0.0307 (5)	−0.0035 (4)	−0.0140 (4)	−0.0039 (4)
C9	0.0391 (6)	0.0228 (5)	0.0351 (6)	−0.0074 (4)	−0.0132 (5)	−0.0083 (4)
C10	0.0360 (6)	0.0325 (6)	0.0268 (5)	−0.0084 (5)	−0.0110 (4)	−0.0098 (4)
C11	0.0310 (5)	0.0312 (5)	0.0197 (4)	−0.0075 (4)	−0.0088 (4)	−0.0040 (4)
C11A	0.0230 (4)	0.0231 (4)	0.0180 (4)	−0.0041 (3)	−0.0055 (3)	−0.0032 (3)
C12	0.0249 (4)	0.0227 (4)	0.0187 (4)	−0.0048 (4)	−0.0076 (3)	0.0018 (3)
C12A	0.0226 (4)	0.0168 (4)	0.0195 (4)	−0.0034 (3)	−0.0065 (3)	0.0017 (3)
S13	0.04278 (16)	0.01844 (12)	0.02660 (13)	−0.01033 (10)	−0.01874 (11)	0.00722 (9)
C13A	0.0170 (4)	0.0157 (4)	0.0179 (4)	−0.0031 (3)	−0.0020 (3)	0.0011 (3)
C14	0.0209 (4)	0.0157 (4)	0.0208 (4)	−0.0053 (3)	−0.0008 (3)	0.0020 (3)
C14A	0.0217 (4)	0.0166 (4)	0.0217 (4)	−0.0074 (3)	−0.0005 (3)	−0.0005 (3)
C15	0.0339 (5)	0.0117 (4)	0.0240 (4)	−0.0009 (3)	−0.0167 (4)	0.0013 (3)
C16	0.0453 (6)	0.0167 (4)	0.0254 (5)	−0.0128 (4)	−0.0162 (4)	0.0049 (4)
C17	0.0271 (5)	0.0251 (5)	0.0229 (4)	−0.0127 (4)	−0.0096 (4)	0.0044 (4)
S19	0.01835 (11)	0.03176 (13)	0.01380 (10)	−0.00181 (9)	−0.00435 (8)	0.00184 (8)
O20	0.0256 (4)	0.0342 (4)	0.0215 (3)	0.0027 (3)	−0.0122 (3)	−0.0053 (3)
O21	0.0277 (4)	0.0619 (6)	0.0180 (3)	−0.0125 (4)	−0.0001 (3)	0.0093 (4)
C22	0.0180 (4)	0.0338 (5)	0.0146 (4)	0.0056 (4)	−0.0031 (3)	−0.0036 (4)
C23	0.0186 (4)	0.0486 (7)	0.0199 (4)	0.0054 (4)	−0.0045 (4)	−0.0059 (4)
C24	0.0257 (5)	0.0564 (8)	0.0206 (5)	0.0174 (5)	−0.0085 (4)	−0.0093 (5)
C25	0.0381 (6)	0.0426 (7)	0.0156 (4)	0.0209 (5)	−0.0028 (4)	−0.0052 (4)
C26	0.0376 (6)	0.0310 (6)	0.0231 (5)	0.0113 (5)	−0.0010 (4)	−0.0044 (4)
C27	0.0252 (5)	0.0290 (5)	0.0222 (4)	0.0066 (4)	−0.0048 (4)	−0.0055 (4)
C28	0.0593 (9)	0.0539 (9)	0.0213 (5)	0.0314 (7)	−0.0067 (5)	−0.0004 (5)
N18	0.0201 (4)	0.0214 (4)	0.0187 (4)	−0.0059 (3)	−0.0051 (3)	0.0011 (3)

### Geometric parameters (Å, º)

**Table:**

C1—C2	1.3730 (16)	S13—C13A	1.7440 (10)
C1—C14A	1.4163 (13)	C13A—C14	1.3689 (13)
C1—H1	0.9500	C14—C14A	1.4152 (14)
C2—C3	1.4124 (16)	C14—H14	0.9500
C2—H2	0.9500	C15—C16	1.5256 (17)
C3—C4	1.3782 (14)	C15—H15A	0.9900
C3—H3	0.9500	C15—H15B	0.9900
C4—C4A	1.4130 (13)	C16—C17	1.5179 (16)
C4—H4	0.9500	C16—H16A	0.9900
C4A—N5	1.3699 (11)	C16—H16B	0.9900
C4A—C14A	1.4158 (13)	C17—N18	1.4748 (13)
N5—C5A	1.3149 (12)	C17—H17A	0.9900
C5A—N6	1.3986 (11)	C17—H17B	0.9900
C5A—C13A	1.4384 (12)	S19—O20	1.4351 (8)
N6—C6A	1.4016 (12)	S19—O21	1.4357 (9)
N6—C15	1.4800 (12)	S19—N18	1.6279 (9)
C6A—N7	1.3156 (12)	S19—C22	1.7623 (11)
C6A—C12A	1.4335 (13)	C22—C27	1.3852 (17)
N7—C7A	1.3704 (12)	C22—C23	1.3974 (14)
C7A—C11A	1.4118 (14)	C23—C24	1.3877 (17)
C7A—C8	1.4133 (14)	C23—H23	0.9500
C8—C9	1.3765 (15)	C24—C25	1.390 (2)
C8—H8	0.9500	C24—H24	0.9500
C9—C10	1.4082 (17)	C25—C26	1.3914 (19)
C9—H9	0.9500	C25—C28	1.5080 (17)
C10—C11	1.3714 (16)	C26—C27	1.3923 (15)
C10—H10	0.9500	C26—H26	0.9500
C11—C11A	1.4158 (13)	C27—H27	0.9500
C11—H11	0.9500	C28—H28A	0.9800
C11A—C12	1.4170 (14)	C28—H28B	0.9800
C12—C12A	1.3660 (13)	C28—H28C	0.9800
C12—H12	0.9500	N18—H18	0.876 (15)
C12A—S13	1.7471 (10)		
			
C2—C1—C14A	120.16 (10)	C14A—C14—H14	119.6
C2—C1—H1	119.9	C14—C14A—C4A	116.65 (8)
C14A—C1—H1	119.9	C14—C14A—C1	123.70 (9)
C1—C2—C3	120.11 (10)	C4A—C14A—C1	119.63 (9)
C1—C2—H2	119.9	N6—C15—C16	113.75 (9)
C3—C2—H2	119.9	N6—C15—H15A	108.8
C4—C3—C2	120.90 (10)	C16—C15—H15A	108.8
C4—C3—H3	119.6	N6—C15—H15B	108.8
C2—C3—H3	119.6	C16—C15—H15B	108.8
C3—C4—C4A	119.82 (10)	H15A—C15—H15B	107.7
C3—C4—H4	120.1	C17—C16—C15	115.55 (8)
C4A—C4—H4	120.1	C17—C16—H16A	108.4
N5—C4A—C4	118.46 (8)	C15—C16—H16A	108.4
N5—C4A—C14A	122.16 (9)	C17—C16—H16B	108.4
C4—C4A—C14A	119.35 (8)	C15—C16—H16B	108.4
C5A—N5—C4A	120.21 (8)	H16A—C16—H16B	107.5
N5—C5A—N6	116.71 (8)	N18—C17—C16	111.15 (9)
N5—C5A—C13A	121.40 (8)	N18—C17—H17A	109.4
N6—C5A—C13A	121.88 (8)	C16—C17—H17A	109.4
C5A—N6—C6A	125.03 (8)	N18—C17—H17B	109.4
C5A—N6—C15	118.16 (8)	C16—C17—H17B	109.4
C6A—N6—C15	116.78 (7)	H17A—C17—H17B	108.0
N7—C6A—N6	115.61 (8)	O20—S19—O21	119.96 (5)
N7—C6A—C12A	121.93 (8)	O20—S19—N18	106.67 (5)
N6—C6A—C12A	122.46 (8)	O21—S19—N18	106.71 (6)
C6A—N7—C7A	119.45 (8)	O20—S19—C22	107.48 (5)
N7—C7A—C11A	122.50 (9)	O21—S19—C22	107.68 (5)
N7—C7A—C8	118.34 (9)	N18—S19—C22	107.82 (4)
C11A—C7A—C8	119.15 (9)	C27—C22—C23	120.77 (10)
C9—C8—C7A	120.19 (10)	C27—C22—S19	119.75 (8)
C9—C8—H8	119.9	C23—C22—S19	119.44 (9)
C7A—C8—H8	119.9	C24—C23—C22	118.36 (13)
C8—C9—C10	120.41 (10)	C24—C23—H23	120.8
C8—C9—H9	119.8	C22—C23—H23	120.8
C10—C9—H9	119.8	C23—C24—C25	121.87 (11)
C11—C10—C9	120.59 (10)	C23—C24—H24	119.1
C11—C10—H10	119.7	C25—C24—H24	119.1
C9—C10—H10	119.7	C24—C25—C26	118.76 (11)
C10—C11—C11A	119.87 (10)	C24—C25—C28	120.26 (13)
C10—C11—H11	120.1	C26—C25—C28	120.97 (15)
C11A—C11—H11	120.1	C25—C26—C27	120.43 (13)
C7A—C11A—C11	119.76 (9)	C25—C26—H26	119.8
C7A—C11A—C12	116.77 (9)	C27—C26—H26	119.8
C11—C11A—C12	123.45 (9)	C22—C27—C26	119.80 (11)
C12A—C12—C11A	120.52 (9)	C22—C27—H27	120.1
C12A—C12—H12	119.7	C26—C27—H27	120.1
C11A—C12—H12	119.7	C25—C28—H28A	109.5
C12—C12A—C6A	118.79 (9)	C25—C28—H28B	109.5
C12—C12A—S13	117.62 (7)	H28A—C28—H28B	109.5
C6A—C12A—S13	123.58 (7)	C25—C28—H28C	109.5
C13A—S13—C12A	102.57 (4)	H28A—C28—H28C	109.5
C14—C13A—C5A	118.69 (8)	H28B—C28—H28C	109.5
C14—C13A—S13	117.28 (7)	C17—N18—S19	117.64 (7)
C5A—C13A—S13	124.04 (7)	C17—N18—H18	112.7 (9)
C13A—C14—C14A	120.87 (9)	S19—N18—H18	109.9 (9)
C13A—C14—H14	119.6		
			
C14A—C1—C2—C3	0.77 (19)	C6A—C12A—S13—C13A	−4.78 (10)
C1—C2—C3—C4	−0.06 (19)	N5—C5A—C13A—C14	−0.73 (14)
C2—C3—C4—C4A	−1.26 (17)	N6—C5A—C13A—C14	178.87 (8)
C3—C4—C4A—N5	−176.04 (10)	N5—C5A—C13A—S13	179.35 (7)
C3—C4—C4A—C14A	1.85 (15)	N6—C5A—C13A—S13	−1.05 (13)
C4—C4A—N5—C5A	179.42 (9)	C12A—S13—C13A—C14	−174.70 (8)
C14A—C4A—N5—C5A	1.59 (14)	C12A—S13—C13A—C5A	5.22 (9)
C4A—N5—C5A—N6	−179.91 (8)	C5A—C13A—C14—C14A	0.45 (14)
C4A—N5—C5A—C13A	−0.29 (13)	S13—C13A—C14—C14A	−179.63 (7)
N5—C5A—N6—C6A	174.38 (9)	C13A—C14—C14A—C4A	0.75 (14)
C13A—C5A—N6—C6A	−5.24 (15)	C13A—C14—C14A—C1	−177.64 (10)
N5—C5A—N6—C15	−3.43 (13)	N5—C4A—C14A—C14	−1.80 (14)
C13A—C5A—N6—C15	176.96 (9)	C4—C4A—C14A—C14	−179.61 (9)
C5A—N6—C6A—N7	−174.51 (9)	N5—C4A—C14A—C1	176.66 (9)
C15—N6—C6A—N7	3.32 (13)	C4—C4A—C14A—C1	−1.14 (14)
C5A—N6—C6A—C12A	5.71 (15)	C2—C1—C14A—C14	178.19 (11)
C15—N6—C6A—C12A	−176.46 (9)	C2—C1—C14A—C4A	−0.16 (16)
N6—C6A—N7—C7A	178.97 (9)	C5A—N6—C15—C16	100.99 (10)
C12A—C6A—N7—C7A	−1.25 (15)	C6A—N6—C15—C16	−76.99 (11)
C6A—N7—C7A—C11A	1.42 (15)	N6—C15—C16—C17	−53.22 (11)
C6A—N7—C7A—C8	−177.56 (10)	C15—C16—C17—N18	−68.56 (11)
N7—C7A—C8—C9	178.05 (11)	O20—S19—C22—C27	13.31 (10)
C11A—C7A—C8—C9	−0.96 (17)	O21—S19—C22—C27	143.85 (9)
C7A—C8—C9—C10	−0.04 (19)	N18—S19—C22—C27	−101.35 (9)
C8—C9—C10—C11	1.32 (19)	O20—S19—C22—C23	−168.77 (8)
C9—C10—C11—C11A	−1.55 (18)	O21—S19—C22—C23	−38.23 (10)
N7—C7A—C11A—C11	−178.25 (10)	N18—S19—C22—C23	76.58 (9)
C8—C7A—C11A—C11	0.72 (15)	C27—C22—C23—C24	0.85 (15)
N7—C7A—C11A—C12	0.08 (15)	S19—C22—C23—C24	−177.05 (8)
C8—C7A—C11A—C12	179.05 (10)	C22—C23—C24—C25	−0.71 (16)
C10—C11—C11A—C7A	0.53 (16)	C23—C24—C25—C26	−0.14 (17)
C10—C11—C11A—C12	−177.68 (11)	C23—C24—C25—C28	−179.19 (10)
C7A—C11A—C12—C12A	−1.76 (15)	C24—C25—C26—C27	0.86 (16)
C11—C11A—C12—C12A	176.50 (10)	C28—C25—C26—C27	179.91 (10)
C11A—C12—C12A—C6A	1.94 (15)	C23—C22—C27—C26	−0.16 (16)
C11A—C12—C12A—S13	−178.82 (8)	S19—C22—C27—C26	177.74 (8)
N7—C6A—C12A—C12	−0.41 (15)	C25—C26—C27—C22	−0.72 (16)
N6—C6A—C12A—C12	179.35 (9)	C16—C17—N18—S19	−167.83 (7)
N7—C6A—C12A—S13	−179.61 (8)	O20—S19—N18—C17	−177.97 (7)
N6—C6A—C12A—S13	0.16 (14)	O21—S19—N18—C17	52.66 (8)
C12—C12A—S13—C13A	176.02 (8)	C22—S19—N18—C17	−62.78 (8)

### Hydrogen-bond geometry (Å, º)

**Table:**

*D*—H···*A*	*D*—H	H···*A*	*D*···*A*	*D*—H···*A*
N18—H18···N5	0.876 (14)	2.243 (15)	2.9973 (12)	144.1 (12)
C15—H15*A*···O20^i^	0.99	2.33	3.1641 (12)	141
C11—H11···O21^ii^	0.95	2.55	3.3933 (13)	149
C15—H15*B*···S13^iii^	0.99	2.86	3.6912 (11)	142
C17—H17*B*···O21^iv^	0.99	2.47	3.3013 (13)	141

Symmetry codes: (i) −*x*+1, −*y*+1, −*z*+2; (ii) *x*, *y*, *z*−1; (iii) −*x*+1, −*y*+1, −*z*+1; (iv) −*x*, −*y*+1, −*z*+2.
